# Effects of indexes of consciousness (IoC1 and IoC2) monitoring on remifentanil dosage in modified radical mastectomy: a randomized trial

**DOI:** 10.1186/s13063-016-1298-0

**Published:** 2016-03-29

**Authors:** Guisheng Wu, Lei Zhang, Xuxiang Wang, Ailan Yu, Zongwang Zhang, Jingui Yu

**Affiliations:** Department of Anesthesiology, Qilu Hospital, Shandong University, Jinan, 250012 China; Department of Anesthesiology, Liaocheng People’s Hospital, Liaocheng, 252000 China

**Keywords:** Index of consciousness, Propofol, Remifentanil, IoC2 monitoring, Unilateral modified radical mastectomy

## Abstract

**Background:**

This study investigated the effects of indexes of consciousness (IoC1 and IoC2) monitoring on remifentanil dosage.

**Methods:**

In this randomized, single-blinded, prospective study, 120 patients undergoing unilateral modified radical mastectomy were randomly assigned to the treatment group (T group, *n* = 60) or control group (C group, *n* = 60). In the T group, patients received both IoC1 (sedation) and IoC2 (analgesia) monitoring, and remifentanil dosages were adjusted by anesthetists according to IoC2. In the C group, remifentanil dosages were adjusted based on the anesthetists’ judgment according to the patients’ vital signs. Remifentanil dose, adjustment frequency, infusion duration, intraoperative adverse events, and quality of anesthetic recovery were compared between the two groups. The primary outcome was the dose of remifentanil.

**Results:**

Compared with the C group, mean remifentanil dosage was significantly higher in the T group (3.8 ± 1.9 versus 3.2 ± 1.2 μg kg^-1^ h^-1^, *P* < 0.05) during the anesthetic period, as was the adjustment frequency of target-controlled infusion (2.9 ± 1.9 versus 2.0 ± 1.2 times/surgery, *P* < 0.05), but there was no difference in infusion duration. Voluntary eye opening, extubation time, and recovery score were not significantly different between the two groups (*P* > 0.05). Total adverse events were significantly reduced in the T group (*P* < 0.05).

**Conclusions:**

IoC1-targeted propofol dosing does not seem to be significantly different to hemodynamic-based monitoring, whereas IoC2 monitoring can increase remifentanil dosage during modified radical mastectomy, but the anesthetic process is more controllable and total adverse events are reduced, which improves the controllability of anesthesia.

**Trial registration:**

Trial registration number: ChiCTR-TRC-13004101, registered on 27 November 2013.

## Background

Depth of anesthesia is commonly assessed in clinical practice by the patient’s clinical signs and symptoms such as blood pressure, heart rate variability, and body movement, but these measures are difficult to convert into a quantitative standard measure. In addition, some technologies are available for objective monitoring of intraoperative pain, but they suffer from limitations and disadvantages [1] and body movements can be used as a surrogate for pain [2]. In recent years, the index of consciousness (IoC) has emerged as a new technique for monitoring depth of anesthesia, which not only objectively measures the patient’s awareness level [3] but also reflects analgesic status [2].

At present, many studies have focused on IoC1 monitoring for sedative depth [4, 5], but only a few studies have focused on IoC2 monitoring for analgesic depth [6]. We hypothesized that IoC2 monitoring would help control the depth of anesthesia. In this study, we applied IoC1 and IoC2 to the use of a sedative agent, propofol, and most importantly, an analgesic agent, remifentanil. Furthermore, we evaluated the effectiveness of IoC monitoring for anesthetic depth (IoC1 and IoC2) versus commonly used vital sign monitoring based on factors such as blood pressure and heart rate.

## Methods

### Study design and patients

This study was a randomized single-blind prospective trial. It was registered with http://www.chictr.org.cn/index.aspx (ChiCTR-TRC-13004101). This study was approved by the Medical Ethics Committee of Liaocheng People’s Hospital (approval number 2013XJS-023). In total, 120 patients who were undergoing elective unilateral modified radical mastectomy under total intravenous anesthesia (TIVA) from 20 January 2014 to 1 June 2014 were consecutively enrolled at the Liaocheng People’s Hospital. Inclusion criteria were: (1) American Society of Anesthesiologists (ASA) class I or II; (2) age 18–65 years old; and (3) body mass index (BMI) 18–30 kg/m^2^. Exclusion criteria were: (1) pregnancy; (2) allergy to the agents used in the study; (3) hypertension; (4) hypotension; (5) tachycardia; or (6) bradycardia. Signed informed consent was obtained from all participants. They were randomized 1:1 to the trial group (T group, *n* = 60) or the control group (C group, *n* = 60).

**Fig. 1:**
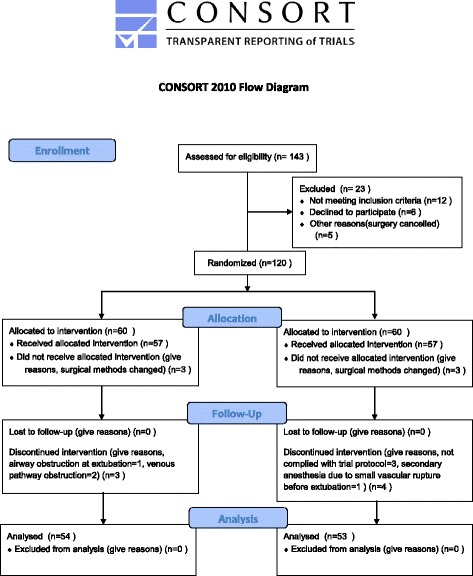
Consort patients flow diagram

Randomization method: 120 opaque and sealed envelopes marked from 1 to 120 were prepared by the researchers, each containing one card written with a random number generated using SPSS 17.0 (IBM, Armonk, NY, USA). If the random number was odd, the patient was allocated to the T group; otherwise the patient was allocated to the C group. Randomization was implemented by a designated individual who did not participate in the subsequent inclusion of patients. The single-blind method was applied to the patients. The T group was monitored using IoC for depth of anesthesia monitoring while the C group was not (Fig. 1).

A patient would be excluded after recruitment if the surgical method was changed peri-operatively or if the anesthetics were finally not used according to the study protocol, for any reason. Other conditions affecting this trial (e.g., airway obstruction, secondary anesthesia given before the patient completely regained consciousness) also led to patient exclusion.

### Anesthesia

After admission to the operating room, the patient was administered 8 ml/kg of Ringer’s solution with an intraoperative maintenance dose of 4 ml kg^-1^ h^-1^. Baseline blood pressure and heart rate were acquired 15 min after admission to the operating room. Both the T and C groups were induced using the Marsh pharmacokinetic model-based target-controlled infusion of propofol. Based on an initial plasma target concentration of 4.0 μg/ml, fentanyl was intravenously injected at 3 μg/kg and cisatracurium at 0.2 mg/kg. Tracheal intubation was performed for mechanical ventilation when satisfactory muscle relaxation was achieved (about 3 min) according to train-of-four (TOF) stimulation evaluation, followed by connection to an anesthetic machine (Drägerwerk AG & Co. KGaA, Lübeck, Germany) for volume-controlled ventilation with a tidal volume of 8 ml/kg, a respiratory rate of 12/min, a respiratory ratio of 1:2, and a pressure of end-tidal carbon dioxide (P_ET_CO_2_) of 35–45 mmHg. At 45 min of surgery, 0.2 mg/kg of cisatracurium was administered for muscle relaxation.

### Anesthesia monitoring

For both groups, non-invasive blood pressure, electrocardiogram (ECG), peripheral oxygen saturation, and P_ET_CO_2_ were routinely monitored. The T group received IoC1 monitoring for depth of sedation and IoC2 monitoring for depth of analgesia (Angel-6000D Multi-parameter Anesthesia Monitor, Shenzhen Weihaokang Medical Technology Co., Ltd, Guangdong, China).

For the generation of IoC [2, 7], the recorded electroencephalogram (EEG) signals are divided into linear and non-linear modules and partitioned using a symbolic dynamics approach. Each part is then labeled by a symbol to convert time series to symbol sequences. Finally, IoC1 is acquired using an adaptive neuro fuzzy inference system based on β ratio and burst suppression rate [8] and IoC2 is derived. The index of depth of sedation, IoC1, ranges from 0 to 99 and is controlled to be within 40–60 during the operative period, with IoC1 > 60 indicating insufficient use of sedative agents while IoC1 < 40 indicates excessive sedation. The index of depth of analgesic, IoC2, ranges from 0 to 99 and is controlled to be within 30–50 during the operative period, with IoC2 > 50 indicating insufficient use of analgesic agents and IoC2 < 30 indicating excessive analgesic effects. Target-controlled infusion is a common, convenient, and accurate administration approach for intravenous anesthesia. In this study, target-controlled infusion was used to record the infusion duration and total dosage of propofol and remifentanil accurately. Previous studies have shown good consistency between IoC and bispectral index [9–14].

During anesthesia, anesthetists adjusted the dosages of propofol and remifentanil according to the changes in IoC1 and IoC2. IoC1, a sedative index, was used as the guide for the adjustment of propofol target concentration, which was increased by 0.5 μg/ml per adjustment when IoC1 > 60 and was increased by 1 μg/ml when body movements were observed. Target concentration was decreased by 0.5 μg/ml per adjustment when IoC1 < 40, with a maintenance value between 40 and 60. IoC2, an analgesic index, was used as the guide for the adjustment of remifentanil target concentration (based on the Minto remifentanil pharmacokinetic parameter set), in which the target concentration was increased by 1 ng/ml per adjustment when IoC2 > 50 but was decreased by 1 ng/ml per adjustment when IoC2 < 30, with the maintenance value between 30 and 50. The C Group was not monitored using IoC, and the doses of propofol and remifentanil were adjusted by the anesthetists according to vital signs such as blood pressure and heart rate so as to control the fluctuation of blood pressure and heart rate within 20 % of baseline values. Meantime, adverse events, such as hypertension, hypotension, tachycardia, and bradycardia, were recorded. The patient’s quality of anesthetic recovery was recorded at the completion of anesthesia.

### Data collection

The following indexes were recorded: (1) general patient data (age, sex, weight, height, BMI, baseline blood pressure, and heart rate); (2) remifentanil (adjustment frequency of target concentration, infusion duration, and mean dosage); (3) propofol (adjustment frequency of target concentration, infusion duration, and mean dosage); (4) frequencies of intraoperative adverse events (hypertension, hypotension, tachycardia, bradycardia, and body movements); and (5) quality of anesthetic recovery [voluntary eye opening, extubation time, awakening score (OAA/S score), and whether the patient showed intraoperative awareness].

Hypertension was defined as intraoperative systolic pressure >160 mmHg, hypotension as intraoperative systolic pressure <90 mmHg, tachycardia as heart rate >90 bpm, and bradycardia as heart rate <45 bpm. Voluntary eye opening time was defined as the interval from stopping the infusion of narcotic drugs to showing voluntary eye movements when calling the patient’s name in a normal voice. The OAA/S score was assessed at the moment of extubation. Intraoperative awareness (using modified Brice questionnaires) was assessed after the patient sobered up.

### Outcomes

The primary outcome was the dose of remifentanil. The secondary outcomes were remifentanil adjustment frequency, infusion duration, intraoperative adverse events, and quality of anesthetic recovery.

### Statistical analysis

The sample size was calculated using a significance level α = 0.05 and test power 1 – β = 0.80. Based on a preliminary trial performed with 20 patients (unpublished data), the mean dosage of remifentanil was 3.0 ± 1.1 μg kg^-1^ h^-1^ in the T group and a significant difference in dosage was defined as a fluctuation ≥20 % compared with the C group. Based on the formula provided by Chow et al. [15] for the calculation of clinical trial sample size, 46 patients were required in each group. However, a total of 120 patients were enrolled in consideration of a dropout rate of 25 %.

All data were analyzed using SPSS 17.0 (IBM, Armonk, NY, USA). Continuous data are shown as mean ± standard deviation (SD). Between-group comparisons were performed using the independent sample *t* test or the rank sum test. Categorical data were compared using the chi-square test. A *P* value <0.05 was considered statistically significant.

## Results

A total of 120 patients with breast cancer were enrolled in January to June 2014 and randomly assigned to the T group (*n* = 60) or C group (*n* = 60). Thirteen patients were excluded. Six patients were excluded from the T group due to a change of the surgical method (*n* = 3), airway obstruction at extubation (*n* = 1), or venous pathway obstruction (*n* = 2). Seven patients were excluded from the C group for non-compliance to trial protocol (*n* = 3), change of the surgical method (*n* = 3), or secondary anesthesia due to a small vascular rupture before extubation (*n* = 1). Therefore, 54 and 53 patients were included in the final analyses for the T and C groups, respectively (Fig. 1).

The two groups were not significantly different in age, height, weight, BMI, systolic pressure, diastolic pressure, and heart rate (all *P* > 0.05) (Table 1).

**Table 1:** Comparison of general data between the two groups (mean ± SD)

	T group (*n* = 54)	C group (*n* = 53)	*P*
Age (years)	47 ± 7	48 ± 8	0.745
Weight (kg)	63 ± 9	63 ± 8	0.983
Height (cm)	160 ± 5	159 ± 4	0.464
BMI (kg/m^2^)	25 ± 3	25 ± 3	0.804
Systolic pressure (mmHg)	130 ± 8	128 ± 10	0.517
Diastolic pressure (mmHg)	77 ± 6	74 ± 7	0.135
Heart rate (bpm)	76 ± 7	75 ± 7	0.291

*BMI* body mass index

As shown in Table 2, remifentanil infusion duration was not significantly different between the two groups (*P* > 0.05), but the adjustment frequency of remifentanil target concentration and mean dosage were different (*P* < 0.05). Comparing the use of propofol and quality of anesthetic recovery, the groups were not significantly different either in adjustment frequency or in mean dosage, with similar voluntary eye opening, extubation time, and awakening score (all *P* > 0.05).

**Table 2:** Comparison of use of remifentanil and propofol and quality of anesthetic recovery between the two groups (mean ± SD)

Item	T group (*n* = 54)	C group (*n* = 53)	*P*
Use of remifentanil			
Adjustment frequency of target concentration (times/surgery)	2.9 ± 1.9^a^	2.0 ± 0.2^a^	0.009^a^
Infusion duration (h)	1.1 ± 0.6	1.3 ± 0.7	0.428
Mean dose (μg kg^-1^ h^-1^)	3.8 ± 1.9	3.2 ± 1.2	0.003
Use of propofol			
Adjustment frequency of target concentration (times/surgery)	3.0 ± 2.0^a^	3.0 ± 1.9^a^	0.444^a^
Infusion duration (h)	1.6 ± 0.5	1.9 ± 0.6	0.523
Mean dosage (μg kg^-1^ h^-1^)	8.8 ± 1.1	8.3 ± 1.0	0.903
Quality of anesthetic recovery			
Voluntary eye opening (min)	5.4 ± 3.1	6.2 ± 2.3	0.782
Extubation time (min)	9.8 ± 6.1	9.3 ± 5.7	0.816
Awakening score	3.9 ± 0.7	4.0 ± 0.9	0.960

^a^ Indicates the results of rank sum test

As shown in Table 3 (intraoperative adverse events), although some apparent differences in frequencies of hypertension (9 % versus 17 %, *P* = 0.24), hypotension (11 % versus 21 %, *P* = 0.17), tachycardia (0 % versus 4 %, *P* = 0.15), bradycardia (17 % versus 8 %, *P* = 0.15), body movement (11 % versus 19 %, *P* = 0.26), and number of patients with adverse events (35 % versus 47 %, *P* = 0.21) could be observed, they were not statistically significant (all *P* > 0.05). Nevertheless, the T group showed significantly less total adverse events (48 % versus 68 %, *P* < 0.05).

**Table 3:** Intraoperative adverse events in the two groups

Item	T group (*n* = 54)	C group (*n* = 53)	*P*
Hypertension	5 (9 %)	9 (17 %)	0.236
Hypotension	6 (11 %)	11 (21 %)	0.173
Tachycardia	0	2 (4 %)	0.150
Bradycardia	9 (17 %)	4 (8 %)	0.149
Body movements	6 (11 %)	10 (19 %)	0.261
Number of patients with adverse events	19 (35 %)	25 (47 %)	0.208
Intraoperative awareness	0	0	1.000
Total adverse events	26 (48 %)	36 (68 %)	0.038

## Discussion

This study investigated the effects of IoC1 and IoC2 monitoring on remifentanil dosage. Results showed that compared with the C group, the mean remifentanil dose was significantly higher in the T group during the anesthetic period, as were the adjustment frequency of target-controlled infusion, but there was no difference in infusion duration. Voluntary eye opening, extubation time, and recovery score were not significantly different between the two groups. Total adverse events were significantly reduced in the T group.

Some studies have shown that pain can induce changes in EEG power [16–18]. Jensen et al. [2] confirmed that IoC1 (qCON) can reliably predict the disappearance of eyelash reflex (or, the disappearance of awareness) during TIVA using propofol and remifentanil. Moreover, with similar concentrations of anesthetics, IoC2 (qNOX) can predict the occurrence of body movements to nociceptive stimuli. In the present study, nociceptive stimuli mainly occurred during anesthesia or surgical procedures such as tracheal intubation/extubation, skin incision, resection of mammary tissues, and axillary lymph node dissection. This study revealed a slight delay (around 1.5–2 min) in IoC2 reduction compared with IoC1 during anesthesia induction, possibly due to a slower action (1 min) of remifentanil versus propofol (30–40 s) or due to the computational errors in the calculation of IoC2 based on IoC1, or both.

Like EEG curves, IoC1 and IoC2 curves constantly change during the maintenance of anesthesia, and they are not smooth. This demonstrates that the so-called depth of anesthesia is essentially a state of the central nervous system that is affected by the interactions between the irritations from nociceptive stimuli and the inhibitory effects of anesthetic agents. In other words, it is a functional state of the central nervous system occurring when surgical stimulation dynamically balances against the control effects of general anesthetics, indicating that IoC1 and IoC2 will still slightly fluctuate due to surgical stimuli although the effect compartment concentrations of propofol and remifentanil or depth of anesthesia is relatively stable. Based on the characteristics of IoC1 and IoC2 and based on our clinical experience, the doses of propofol and remifentanil should be changed when both IoC1 and IoC2 exceed or are lower than their reference ranges for over 2 min, or when both indexes exceed more than 20 within 1 min, to avoid frequent adjustments of infusion speed. Nevertheless, trials should look specifically at the most optimal protocols for changes in anesthetic doses using IoC1 and IoC2.

In the C group, the doses of propofol and remifentanil were adjusted based on the patient’s baseline blood pressure and heart rate to make them fluctuate within 80–120 % of baseline values during surgery. Although the adjustment frequency and mean dose of remifentanil were higher in the T group than in the C group, the T group showed more stable hemodynamics, and smaller frequencies of hypertension, hypotension, and adverse events. In the T group, a relatively high incidence of bradycardia may have resulted from the side effects of slowing down the heart rate by remifentanil, but as an ultra-short-acting opioid analgesic, remifentanil does not affect the patient’s awakening. During maintenance of anesthesia, the effect compartment concentration of propofol was kept within 2.5–6.0 μg/ml in both groups, but remifentanil showed a huge variation among patients, with the effect compartment concentrations being within 0–7 ng/ml.

In TIVA, the sedative agent (propofol) and analgesics (fentanyl and remifentanil) do not show muscle relaxation effects, and there is a need for administration of muscle relaxants. During surgery, the muscle relaxant cisatracurium was administered at 45 min with 0.2 mg/kg. In the T group, seven patients showed body movements before muscle relaxants were administered, although their hemodynamic indexes reached the lower limits, and interestingly, three patients showed body movements even with a low yet normal index of depth of anesthesia, indicating that the reliability of IoC monitoring for depth of anesthesia in predicting body movements should be further studied in the future.

In the present study, five patients who underwent preoperative chemotherapy showed higher IoC1 and IoC2 than the reference ranges for about 75 % of the time (among whom some showed light anesthesia with IoC1 and IoC2 reaching 73–88, which almost indicated regained consciousness), although their effect compartment concentrations of propofol and remifentanil were 3.5–5.5 μg/ml and 2–5 ng/ml, respectively, and their hemodynamic indexes were within the reference ranges. According to previous studies, preoperative chemotherapy can damage liver and renal functions and the nervous system [19, 20], and chemotherapy drugs can boost the effects of and reduce the use of anesthetic drugs. However, in this study, the doses of anesthetics were higher in the patients with breast cancer who underwent preoperative chemotherapy. They also showed faster metabolism of muscle relaxants and faster recovery of spontaneous respiration, inconsistent with previous studies. Since only a few patients (17 in the C group and 12 in the T group) with preoperative chemotherapy were included in this study, the above inconsistency is not explainable and thus, should be further validated by other studies. Unlike bispectral index monitoring, the monitor used in this study does not require special electrodes, which reduces medical expenditures and promotes clinical propagation and application. Normally, the EEG amplitude is within 0–200 μV, about 1/400 of cardiac electrical activities; hence, compared with ECG, it is more easily affected by widely used high-frequency electrical equipment such as an electrocoagulation electrotome. We chose patients undergoing mastectomy because the electrotome used for surgery is low in power and distant from the site of data collection and as a result, the effects of interference are smaller. Nonetheless, this disadvantage may still affect its further clinical application and some improvements should be made on surgical site selection, materials for production of the monitor, and filtering clutter, among others.

This study suffers from some limitations. The sample size was small. We only studied the application of IoC monitoring to TIVA and thus its effectiveness with inhaled anesthesia is unclear. In addition, only female patients were included in our study, but the possible differences of pharmacokinetics with male patients should be considered. Fentanyl was used for intubation, but the same dose was used in both groups; therefore, it should not influence the comparison between the two groups. Importantly, propofol administration was not standardized and was based on IoC1, therefore introducing variability that could mask the real effects of IoC2 monitoring. In addition, standard monitoring parameters, such as bispectral index, should be included in future studies to correlate the parameters with IoC2. Finally, some confounding factors that may affect our results have not been controlled and additional studies are necessary to examine the impact of neoadjuvant chemotherapy on IoC2.

## Conclusions

In summary, EEG anesthesia depth index (IoC) monitoring during TIVA is safe and effective for patients with breast cancer. IoC1-targeted propofol dosing does not seem to be significantly different to hemodynamic-based monitoring, whereas the application of IoC2 might be used to guide the use of remifentanil. It might reduce the occurrence of adverse events and keep hemodynamics more stable, but its reliability in predicting body movements as well as the anti-interference ability should be further improved.

## Abbreviations

BMI: body mass index
ECG: electrocardiogram
EEG: electroencephalogram
IoC: index of consciousness
P_ET_CO2: pressure of end-tidal carbon dioxide
SD: standard deviation
TIVA: total intravenous anesthesia
